# Supplementary material for: Nanostructured and Spiky Gold Shell Growth on Magnetic Particles for SERS Applications
Source: Nanomaterials (Basel). 2020 Oct 27;10(11):2136. doi: 10.3390/nano10112136 (PMC7693944; doi:10.3390/nano10112136)
Supplement: Supplementary file 1 [file nanomaterials-10-02136-s001.pdf]

Supporting information

Nanostructured and spiky gold shell growth on magnetic particles for SERS applications

Erin E. Bedford [1,2], Christophe Méthivier [1], Claire-Marie Pradier [1], Frank Gu [2] and Souhir Boujday [1,*]

[1] Laboratoire de Réactivité de Surface (LRS), CNRS, UMR 7197, Sorbonne Université, 4 place Jussieu, F-75005 Paris, France; eebedford@gmail.com (E.E.B.); christophe.methivier@sorbonne-universite.fr (C.M.); claire-marie.pradier@upmc.fr (C.-M.P.); souhir.boujday@sorbonne-universite.fr (S.B.)

[2] Department of Chemical Engineering and Waterloo Institute for Nanotechnology, University of Waterloo, 200 University Ave. W., Waterloo, Canada; eebedford@gmail.com (E.E.B.); frank.gu@uwaterloo.ca (F.G.)

* Correspondence: souhir.boujday@sorbonne-universite.fr (S.B.); Tel.: +33-1-4427-6001

Experimental conditions for SERS measurement

First set: influence of silicon-iron oxide core functionalization: In the first set of experiments, samples for surface-enhanced Raman scattering (SERS) were prepared by adding 50 µL of particles dispersed in CTAB to 3 mL of Millipore water; this amount was chosen because it was the minimum amount required for signal saturation. Particles were initially dispersed in 1 mM CTAB (final CTAB concentration of 16.7 µM) and varying amounts of the Raman probe, 2-mercaptopyrimidine (MPym), were added.

Second set: influence of bath conditions: In the second set of experiments, samples were also prepared by adding 50 µL of particles dispersed in CTAB to 3 mL of Millipore water, but particles were initially dispersed in 10 mM CTAB (final CTAB concentration of 167 µM), which resulted in a signal large enough to be compared without the use of an additional Raman probe.

UV-Visible spectra during gold seed attachment and upon shell growth

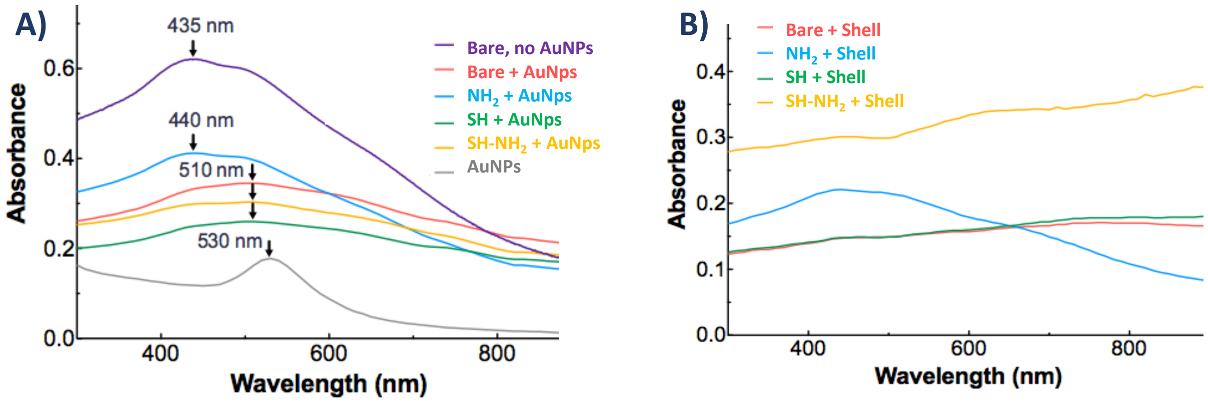

Figure S1. UV-Vis spectra of A) particles before and after gold seeds binding, B) spiky particles.

Magnetic properties

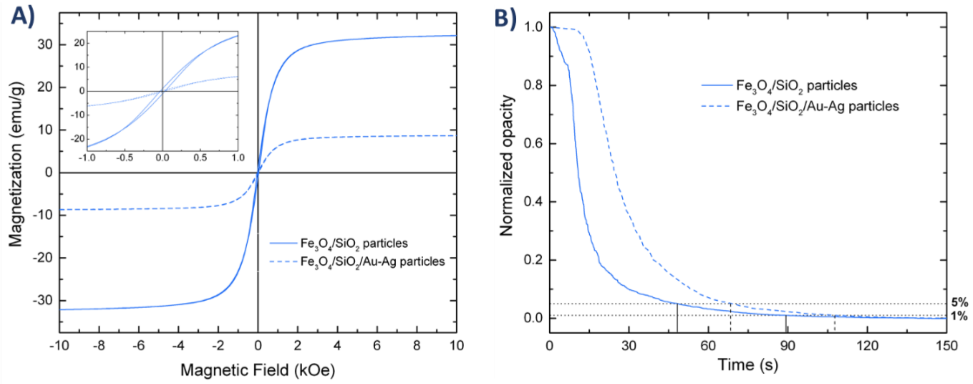

Figure S2: A- Magnetization curves of silica-coated iron oxide particles before (solid) and after (dashed) gold/silver shell coating. The inset shows the small amount of hysteresis occurring at low magnetic fields. B-Measured opacity over time for water dispersions of particles at 1 mg Fe_3O_4/SiO_2 particles/mL (greater mass upon gold coating) in a 45 T/m gradient, before (solid) and after (dashed) gold shell coating

The magnetization curve (inset in Figure S2) also shows that particles have low remanent magnetization; for the silica-coated iron-oxide particles, the remanent magnetization is 1 emu/g and for the gold-coated particles, the value is 0.25 emu/g, suggesting that the particles do not meet the formal definition of superparamagnetism, but with despite such a low value, they do exhibit superparamagnetic behavior in practical applications.

Table S1: Separation times of particles, determined based on time required to reach 5% and 1% of initial opacity

	**Separation time (seconds)**	
	5% opacity	*1% opacity*
**Fe_3O_4/SiO_2**	48	89
**Fe_3O_4/SiO_2/Au-Ag**	68	108

SERS detection of MPym

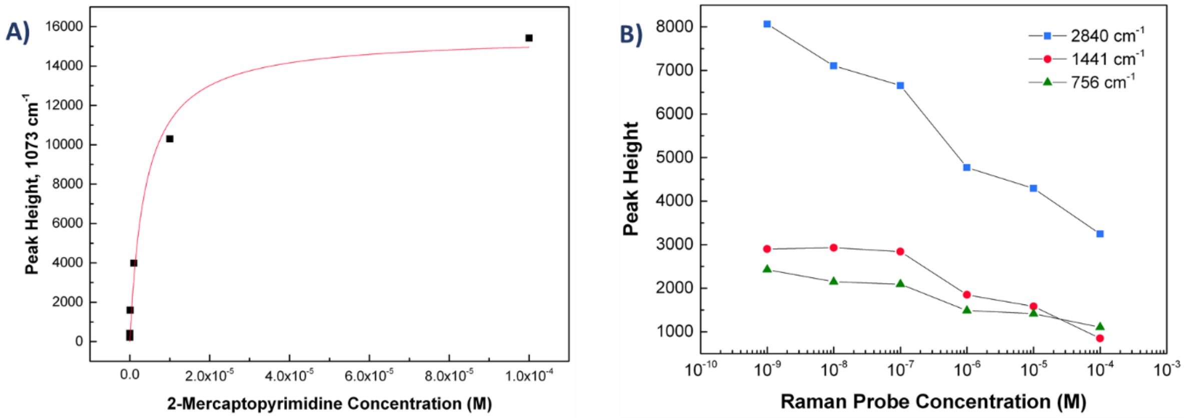

Figure S3: A- Change in peak height of Raman signal corresponding to the Raman reporter (MPym) at different concentrations. A fit to a Langmuir model (red line) shows saturation behavior (R^2 = 0.98), B- Change in peak heights corresponding to CTAB at different Raman reporter (MPym) concentrations.
